# IgG4-related disease presenting as recurrent scleritis combined with optic neuropathy

**DOI:** 10.1186/s12886-020-01774-6

**Published:** 2021-01-05

**Authors:** Su Jin Kim, Seung Uk Lee, Min Seung Kang, Jung Hyo Ahn, Jonghoon Shin, Choul Yong Park, Ji Eun Lee

**Affiliations:** 1 Department of Ophthalmology, Pusan National University Yangsan Hospital, Pusan National University School of Medicine, 20 Geumo-ro, Mulgeum-eup, Yangsan-si, Gyeongsangnam-do 50612 South Korea; 2 Research Institute for Convergence of Biomedical Science and Technology, Pusan National University Yangsan Hospital, Yangsan, South Korea; 3 Department of Ophthalmology, School of Medicine, Kosin University, #34 Amnam-dong, Seo-gu, Busan, 602-702 South Korea; 4 Department of Ophthalmology, Dongguk University Ilsan Hospital, #27 Dongguk-ro, Ilsandong-gu, Goyang, 10326 South Korea; 5 Sensory Organ Research Center, Dongguk University, Goyang, South Korea

**Keywords:** Extraocular muscle, IgG4-related disease, Optic neuropathy, Recurrent scleritis

## Abstract

**Background:**

We report a case of atypical presentation of IgG4-related disease (IgG4-RD) with recurrent scleritis and optic nerve involvement.

**Case presentation:**

A 61-year-old male presented with ocular pain and injection in his left eye for 2 months. Ocular examination together with ancillary testing led to the diagnosis of scleritis, which relapsed in spite of several courses of steroid treatment. After cessation of steroid, the patient complained of severe retro-orbital pain and blurred vision. His best corrected vision was count finger, the pupil was mid-dilated and a relative afferent pupillary defect was found. Funduscopic examination demonstrated disc swelling. Magnetic resonance imaging (MRI) showed enhancing soft tissue encasing the left globe, medial rectus muscle and optic nerve. Systemic work-up revealed multiple nodules in right lower lung and a biopsy showed histopathological characteristics of IgG4-RD. Long-term treatment with corticosteroids and a steroid-sparing agent (methotrexate) led to significant improvement in signs and symptoms with no recurrence for 2 years.

**Conclusions:**

This case highlights the significance of IgG4-RD in the differential diagnosis of recurrent scleritis. IgG4-RD may cause optic neuropathy resulting in visual loss. Early diagnosis and proper treatment can prevent irreversible organ damage and devastating visual morbidity.

## Background

IgG4-related disease (IgG4-RD) is a newly identified clinical entity characterized by an IgG4-positive multi-organ lymphoplasmacytic infiltrate, associated with fibrosis or sclerosis [1–3]. Since IgG4-RD was first described in conjunction with autoimmune sclerosing pancreatitis, the disorder has been expanded to incorporate several previously distinct inflammatory conditions [4–6].

Ophthalmic involvement predominantly affects the lacrimal gland and typically presents as idiopathic orbital inflammation and chronic sclerosing dacryoadenitis [7, 8]. Here, we report an atypical case of IgG4-RD revealed by chronic relapsing scleritis with optic nerve sheath and extra-ocular muscle (EOM) involvement and confirmed by pathological analysis from the pulmonary nodules.

## Case presentation

**Fig. 1:**
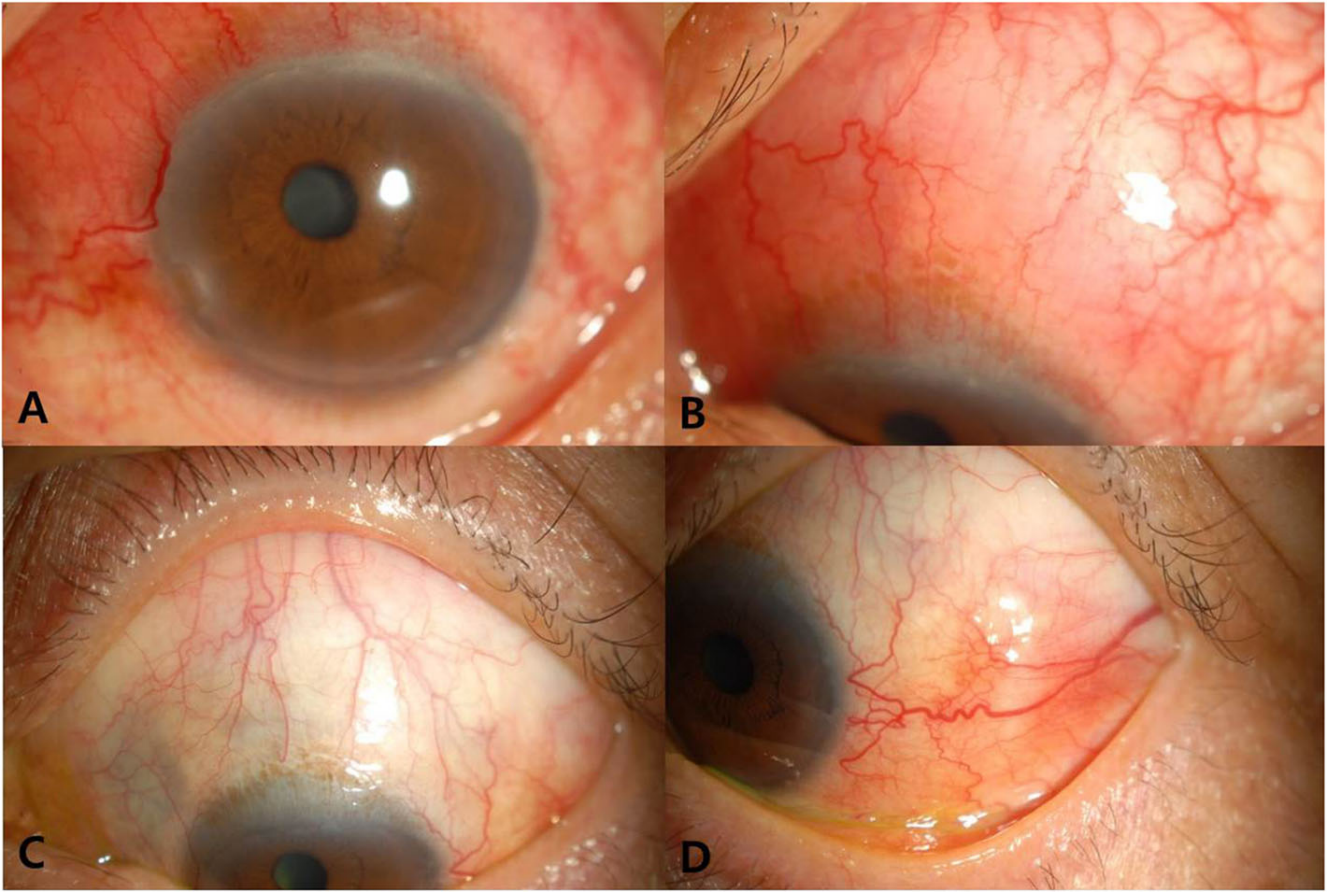
Clinical picture of the patient demonstrating scleritis. Slit lamp examination showed a localized inflamed superonasal bulbar conjunctival swelling in the left eye (**a** & **b**). During steroid treatment, superonasal lesion had been resolved (**c**) but new lesion was developed on the temporal area (**d**)

A 61-year-old man presented with unilateral ocular pain and injection for 2 months. His best corrected visual acuity was 20/20 bilaterally and intraocular pressures were normal. Slit lamp examination showed a localized inflamed superonasal bulbar conjunctival swelling in the form of scleritis in his left eye (Fig. 1a and b). Normal fundus with no choroidal folds or serous retinal detachment was found. On peripheral blood tests, full blood count, leucocyte count, erythrocyte sedimentation rate and C-reactive protein were normal. Testing for antinuclear antibodies, antineutrophil cytoplasmic antibodies, rheumatoid factor, anti-dsDNA IgG, angiotensin converting enzyme and HLA-B27 were normal or negative. The patient was diagnosed with anterior scleritis and treated with topical steroid (fluometholone 4 times a day) and oral prednisolone (30 mg once a day). During tapering the steroid, previous lesion subsided (Fig. 1c), but newly developed lesion was found on the temporal area of the left eye at a dose of 10 mg/d of prednisolone (Fig. 1d). The relapse and remission was repeated for 6 months in spite of several courses of steroid treatment.

**Fig. 2:**
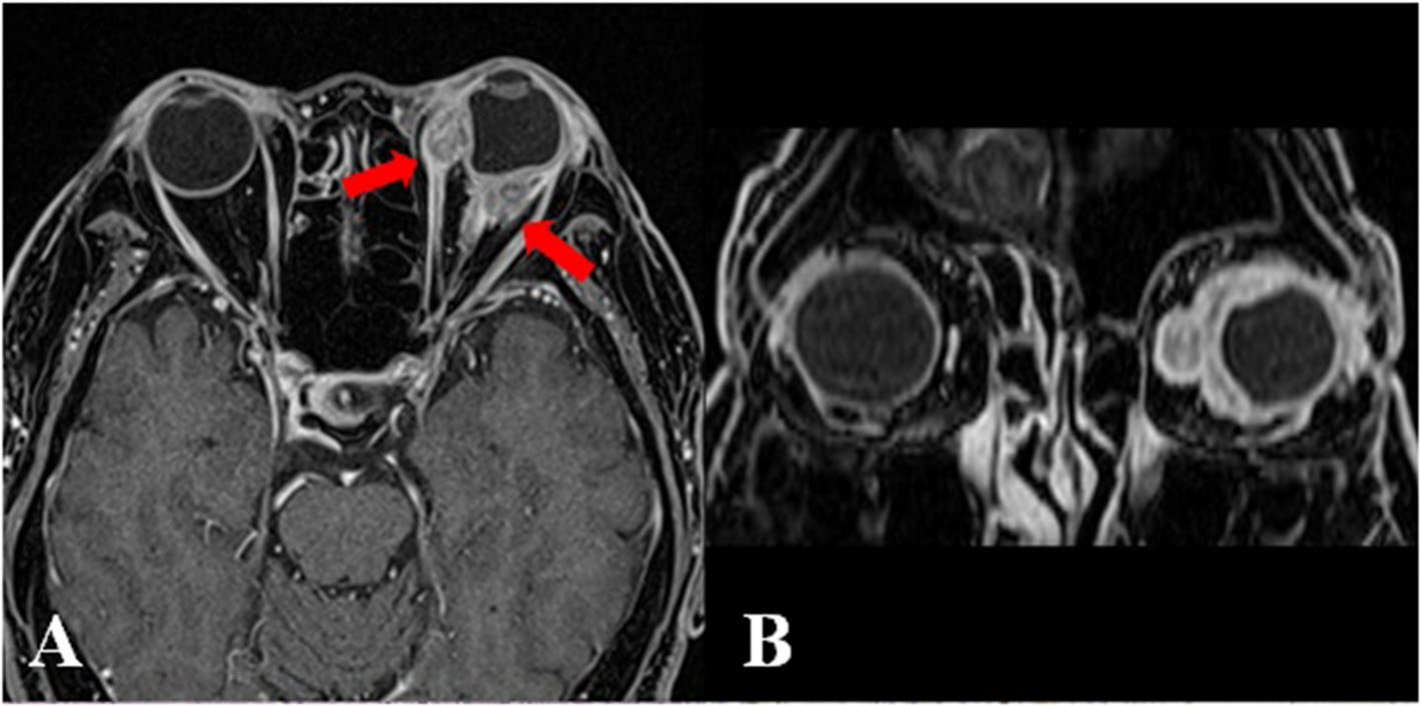
Orbit MRI demonstrated enhancing soft tissue encasing the left globe (**a** & **b**) with encasement of left medial rectus muscle and optic nerve (red arrows)

**Fig. 3:**
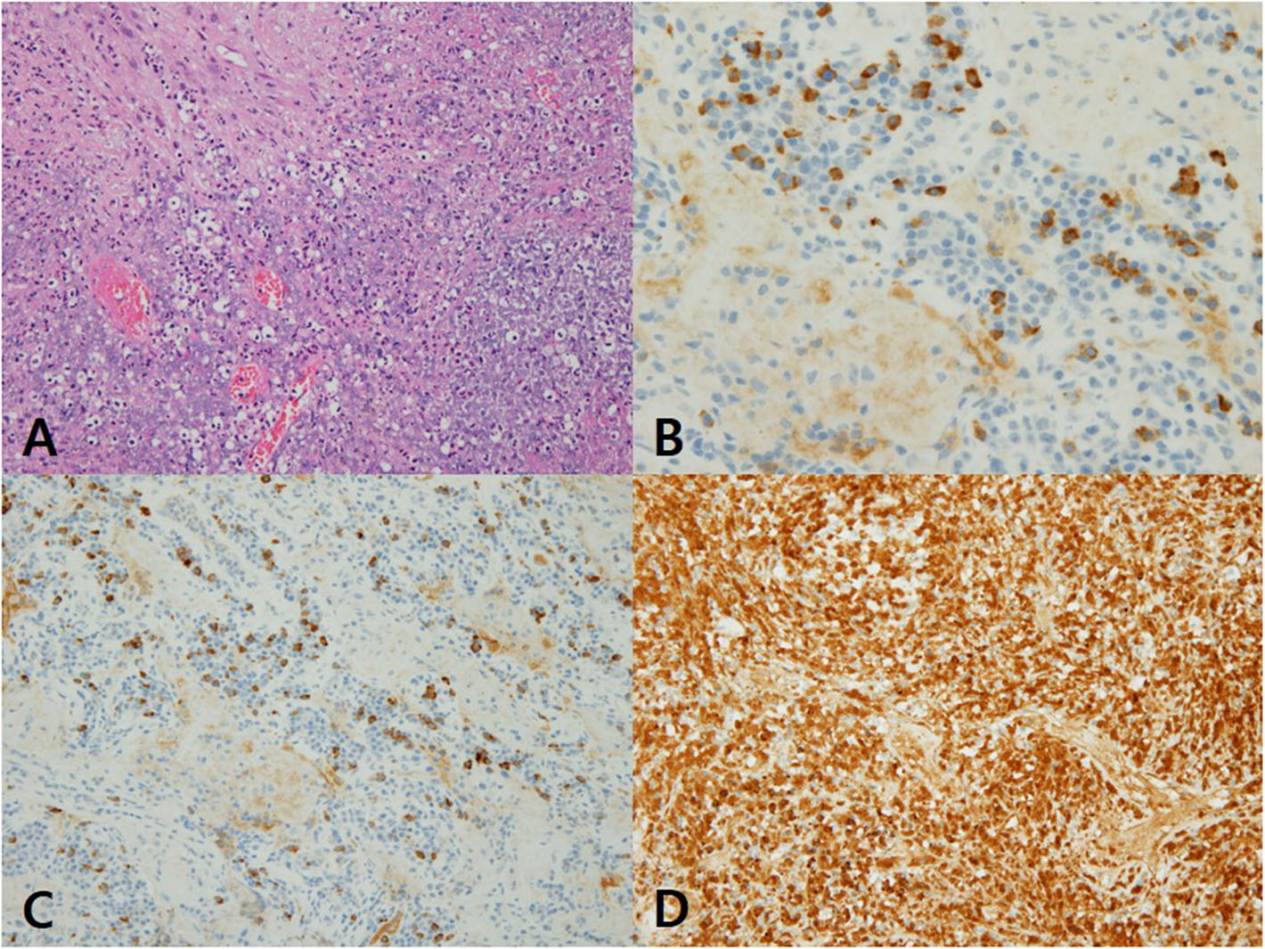
Microscopic examination of the pulmonary nodule revealed necrotizing inflammation, dense fibrosis, marked lymphoplasmacytic infiltration, and increased IgG4+ plasma cells (**a**, × 200, Hematoxylin and eosin staining). Immunohistochemical staining showed an increased number of IgG4+ plasma cells (**b**, more than 50 IgG4 cells per HPF, × 400) and an elevated IgG4+/IgG+ ratio (**c** and **d**, a ratio of IgG4+ to IgG+ cells of 46.5%)

One week after the cessation of steroid, he complained of severe retro-orbital pain and blurred vision in his left eye. His left vision was decreased into count finger. The pupil was mid dilated and a relative afferent pupillary defect was found. Funduscopic examination demonstrated vitritis and disc swelling in the left eye. Orbit magnetic resonance imaging (MRI) demonstrated enhancing soft tissue encasing the left globe, where medial rectus muscle and optic nerve were enlarged and enhanced with scleral thickening (Fig. 2). On systemic evaluation, computed tomography (CT) scan of the chest revealed multiple clustered nodules in right lower lung (RLL) with tubular shadow and multiple small mediastinal lymph nodes. Video-assisted thoracoscopic surgery (VATS) biopsy was performed and immunohistochemical stain against IgG4 demonstrated that the number of IgG4-positive plasma cell were elevated to > 50 cells per high-power field (Fig. 3b). In addition, an IgG4/IgG plasma cell ratio of 46.5% was observed (Fig. 3c and d). Serum IgG4 level was elevated at 210.7 mg/dl.

The patient was diagnosed as IgG4-RD and began intravenous (IV) injection of hydrocortisone 50 mg tid for 1 week, followed by IV methylprednisolone 60 mg for 4 days. He was treated with 25 mg of oral methylprednisolone daily for 2 weeks, the dose was gradually reduced by 5 mg every 2 weeks and maintained methotrexate 10 mg once a week. Long-term treatment with corticosteroids and a steroid-sparing agent (methotrexate) led to significant improvement in signs and symptoms. Visual acuity was improved into 20/40 and no recurrence was observed after 2 years of follow-up.

## Discussion and conclusions

In 2011, comprehensive diagnostic criteria for IgG4-RD was defined as meeting two or more and including 1) of the following: 1) clinical examination shows characteristic diffuse/localized swelling or masses in single or multiple organs, 2) hematologic examination shows elevated serum IgG4 concentrations (≥135 mg/dl), 3) histopathologic examination shows: marked lymphocyte and plasmacyte infiltration and fibrosis, infiltration of IgG4+ plasma cells: ratio of IgG4+ to IgG + cells > 40% and > 10 IgG4+ plasma cells/HPF [8]. In this study, the patient had recurrent scleritis with soft tissue mass encasing the left globe and pulmonary nodules. Serum IgG4 level was 210.7 mg/dl and histologic examination of the lung nodule showed infiltration of IgG4+ plasma cells. Therefore, this patient fulfills three of diagnostic criterias and diagnosed as definite IgG4-RD.

IgG4-related ophthalmic disease (IgG4-ROD) forms a significant proportion of what has previously been labeled idiopathic orbital inflammation or reactive lymphoid hyperplasia. The lacrimal gland is most commonly involved but other orbital structures affected include the EOM, eyelid, the supraorbital and infraorbital nerves [7–9]. IgG4-ROD is a new and rare entity, which can be difficult to diagnose, especially in case of atypical features such as scleritis and conjunctival infiltration [10, 11]. There are a few reports of scleritis caused by IgG4-RD. Moreover, two IgG4-RD cases involving the sclera were misdiagnosed as intraocular tumor [12, 13], and Ohno et al. [12] reported as choroidal tumor and enucleation of the eyeball was performed. Contrary to the previous studies, our patient could have had the opportunity to avoid the discouraging surgery by simply performing serological tests.

Long diagnostic delay in IgG4-RD, between 3.8 years and 7.5 years in other reports [10, 14], is responsible for permanent sequelae due to extensive fibrosis. In this case, although wax and wane of scleritis had been repeated for 6 months, IgG4-RD was confirmed through systemic work up and biopsy after the orbital mass encasing the optic nerve and medial retus muscle had developed. Inflammatory tissue biopsies and serum IgG4 levels are the only warrant of a timely and accurate diagnosis, and fluorodeoxyglucose-positron emission tomography (FDG-PET) scan is the most effective radiological test to investigate the presence of other organs involvement and to find the most accessible biopsy sites [15]. In our patient, pulmonary nodules in RLL was found at the chest CT scan and VATS biopsy revealed marked lymphoplasmacytic infiltration with IgG4+ plasma cells. We were unable to biopsy the orbital mass due to their proximity to the optic nerve. MRI finding did not show the specific feature of infraorbital nerve enlargement, differentiating optic nerve sheath inflammation of IgG4-ROD from other orbital disease [16]. However, histopathological findings of the pulmonary nodules, elevation of serum IgG4, and improvement of symptoms immediately after steroid administration are supportive of the diagnosis [2, 8, 17].

Corticosteroids are typically the first line of therapy for IgG4-RD. Most patients respond well to oral glucocorticoid within several weeks, but the lesion sometimes recurs during steroid tapering or following the withdrawal. Immunosuppressive agents such as methotrexate, azathioprine, cyclophosphamide, 6-mercaptopurin and bortezomib have been used in IgG4-ROD for steroid-sparing effect. Since lymphoplasmocyic infiltration with CD20+ cells is one of the hallmarks of the disease, rituximab (an anti-CD20 monoclonal antibody) may be a treatment of choice, with a response rate of 90% in refractory cases [18, 19]. Although treatment was started within 1 week of developing visual disturbance in this case, visual acuity was not recovered completely similar to other reports [17, 20] because of the involvement of optic nerve. The optic neuropathy leading to vision impairment may be due to a compressive or inflammatory process or both.

Many systemic diseases such as the autoimmune connective tissue diseases of rheumatoid arthritis, systemic lupus erythematosus, sero-negative spondylarthropathies and vasculitides such as granulomatosis with polyangiitis and polyarteritis nodosa had been reported to cause scleritis [21]. Recently, IgG4-RD has been introduced as an emerging cause of idiopathic scleritis.

Here, we presented a rare case of atypical presentation of IgG4-ROD with recurrent scleritis, optic neuropathy and EOM involvement. In cases of scleritis, the differential diagnosis should include IgG4-RD and serum IgG4 level is warranted as a part of the initial assessment of scleritis. Although this is the most common laboratory test performed for the diagnosis of IgG4-RD, serum IgG4 levels are normal in 30 ~ 40% patients, indicating a reduced sensitivity [22]. Therefore, if there are enlarged or inflamed tissues, proper pathological analysis should be performed to confirm the diagnosis of IgG4-RD.

IgG4-RD should be suspected in the face of any chronic inflammatory ophthalmological conditions and can be added as a novel cause of scleritis. It is important to promptly and accurately recognize IgG4-RD to allow commencement of appropriate treatment and to improve prognosis of patients. Early diagnosis and proper treatment can prevent irreversible organ damage and devastating visual morbidity.

## Data Availability

All data have been presented within the manuscript and in the form of images.
